# Burnout and Cardiovascular Risk in Healthcare Professionals During the COVID-19 Pandemic

**DOI:** 10.3389/fpsyt.2022.867233

**Published:** 2022-04-04

**Authors:** Fayeza Alameri, Noura Aldaheri, Sarah Almesmari, Manea Basaloum, Nouf Albdulrahman Albeshr, Mecit Can Emre Simsekler, Nnamdi Valbosco Ugwuoke, Murat Dalkilinc, Mai Al Qubaisi, Luciana Aparecida Campos, Wael Almahmeed, Eman Alefishat, Hashel Al Tunaiji, Ovidiu Constantin Baltatu

**Affiliations:** ^1^Zayed Military Hospital, Abu Dhabi, United Arab Emirates; ^2^College of Engineering, Khalifa University, Abu Dhabi, United Arab Emirates; ^3^Department of Pharmacology, College of Medicine and Health Sciences, Khalifa University, Abu Dhabi, United Arab Emirates; ^4^Department of Public Health and Epidemiology, College of Medicine and Health Sciences, Khalifa University, Abu Dhabi, United Arab Emirates; ^5^Center of Innovation, Technology and Education (CITE) at Anhembi Morumbi University - Anima Institute, São José dos Campos, Brazil; ^6^Heart and Vascular Institute - Cleveland Clinic, Abu Dhabi, United Arab Emirates; ^7^Department of Biopharmaceutics and Clinical Pharmacy, Faculty of Pharmacy, The University of Jordan, Amman, Jordan; ^8^Center for Biotechnology, Khalifa University, Abu Dhabi, United Arab Emirates; ^9^Academic and Research Committee, Zayed Military University, Abu Dhabi, United Arab Emirates

**Keywords:** cardiovascular risk (CV risk), emotional exhaustion (EE), depersonalization (DP), work stress, REM sleep, sleep alterations

## Abstract

**Introduction:**

The objective of this study was to investigate the psychosocial and cardiovascular markers in healthcare professionals during the COVID-19 pandemic.

**Methods:**

This was a STROBE compliant, blended exploratory study. Residents, staff physicians, nurses, and auxiliary healthcare professionals from both inpatient and outpatient medicine services were recruited using a planned random probability sample. The Maslach Burnout Inventory (MBI), Fuster-BEWAT score (FBS), and socio-demographic factors, as well as sleep quality, were studied. The correlations between burnout severity and cardiovascular risk were examined using multivariable linear regression models adjusted for confounding variables, such as sociodemographic and anthropometric characteristics.

**Results:**

The regression analysis with FBS as the outcome showed a negative association between cardiovascular health and emotional exhaustion [Coef.(95%CI): −0.029 (−0.048, −0.01), *p* = 0.002]. The higher the emotional exhaustion the lower the cardiovascular health. Further, the model showed a positive association between personal accomplishment and cardiovascular health [Coef.(95%CI): 0.045 (0.007, 0.082), *p* = 0.02]. Emotional exhaustion was significantly positive correlated with REM sleep and light average (Spearman’s rank correlation: 0.37 and 0.35, respectively, with *P* < 0.05).

**Conclusion:**

The data from this study show that healthcare practitioners who are with burnout and emotional exhaustion have an elevated cardiovascular risk, however, causality cannot be determined. As an adaptive response to stressful situations, REM sleep increases. The findings of this study may be relevant in creating preventive strategies for burnout and cardiovascular risk reduction or prevention.

**Clinical Trial Registration:**

[www.ClinicalTrials.gov], identifier [NCT04422418].

## Introduction

The coronavirus disease 2019 (COVID-19) pandemic has presented healthcare systems with new and unanticipated obstacles. This quickly changing scenario has a significant impact on healthcare professionals (1). During the epidemic, all healthcare modalities encountered similar obstacles, such as limited expert staff availability and the possibility of peri-procedural transmission of SARS-CoV-2 between patients and staff (2). Managing the unforeseeable scenario of the COVID-19 pandemic posed an unprecedented challenge to healthcare leadership, who had to act quickly to restructure and deliver the key resources and information staff required to manage throughout this health crisis (3).

The COVID-19 pandemic resulted in high incidence of anxiety, depression and burnout syndrome, and mental health disorders among nurses and physicians (4–7). The severe COVID-19 outbreak has had a significant impact on the mental health of medical and nursing professionals (8). A recent study in the Middle East Region found that healthcare professionals experienced psychological distress during the outbreak (9). These factors contribute to physician burnout, which is already a significant problem in the healthcare industry (10, 11).

Long-term job stress can cause burnout, persistent weariness, anxiety symptoms, and health problems (12, 13). According to Maslach and Leiter (14), “Burnout is a psychological syndrome emerging as a prolonged response to chronic interpersonal stressors on the job” (14). According to Karasek and Theorell Job Demands Control Support Model (15), there is a relationship between cardiovascular risk factors and burnout, which is mediated by life and job stressors (16). Occupational stress and burnout can cause persistent fatigue symptoms, as well as an increased risk of cardiovascular disease (17). Furthermore, total occupational burden has been linked to cardiovascular risk in physicians (18). Stress and unfavorable psychosocial working conditions may exacerbate cardiovascular disease in the physician occupational group (19). Overwork-related mortality can be linked to cerebrovascular illnesses (17), whereas induced Takotsubo cardiomyopathy has been linked to work-related stress (20, 21).

The goal of this project was to investigate the relationship between burnout and cardiovascular disease in healthcare professionals using multivariable linear regression models that were adjusted for relevant confounding variables such as socio-demographics and anthropometrics, as well as traditional CVD risk factors.

## Materials and Methods

### Ethics

Ethical approval was obtained from the Abu Dhabi COVID19 Research IRB Committee of the Department of Health-Abu Dhabi (DOH/NCVDC/2020/1052) and Emirates IRB for COVID Research Committee (DOH/CVDC/2020/1246). Informed consent was obtained before accepting to complete the online questionnaires (22, 23).

### Design and Setting

This was an exploratory observational cross-sectional study that covered Hospitals in Abu Dhabi capital of the United Arab Emirates. The study was carried out from July to November 2020, during the second wave of the COVID-19 pandemic, when medical countermeasures were being implemented (24, 25). The STROBE–The EQUATOR Network guidelines were followed to design the protocol of this study (26).

### Study Participants Recruitment and Sample Size

The online survey was distributed to a cohort of healthcare professionals from Abu Dhabi hospitals and healthcare institutions via institutional mass email addressed by either a campus office or the researcher (27, 28). Inclusion criteria were all residents, staff physicians, nurses and auxiliary healthcare professionals from both inpatient and outpatient medical services who agreed to be a part of the study. Exclusion criteria is not willing to complete a written consent form. Potential participants in the study were recruited through institutional mass email, which is sent by either a campus office or the researcher (27). The study uses random probability sampling with each population member having a non-zero chance of being selected. Since our study was sponsored by academic and governmental agencies, we expected to have good response rates for mass email recruitment (29). Electronic surveys are widely used for collecting data in an efficient and timely manner (30) and therefore were used for Maslach Burnout Inventory (MBI), Fuster-BEWAT score (FBS) and socio-demographic characteristics.

### Outcomes

Primary outcomes were (I) Burnout through MBI emotional exhaustion (EE), depersonalization (DP), and personal accomplishment (PA) subscale scores; (II) Cardio-vascular risk through FBS (31, 32). The MBI is the most commonly used standard for assessing burnout in healthcare professionals. This validated instrument contains 22 elements that include an overall measure of burnout as well as three distinct burnout domains: personal accomplishment, emotional exhaustion, and depersonalization (33). The FBS is a cardiovascular health metric consisting of 5 modifiable risk factors: blood pressure, exercise, weight, alimentation, tobacco. Notably, the Fuster-BEWAT and the AHA cardiovascular risk scores predicted the presence and extent of subclinical atherosclerosis (32) and left ventricular hypertrophy classification (34) with similar accuracy. The FBS is a simplified method that does not require laboratory tests and therefore can be applied in primary care settings or communities. Secondary outcomes were (III) sleep quality through wearable monitoring technology (Polar Ignite) (35).

### Data Integration and Management

The collection and management of data consisted of 3 distinct steps, as described in the published protocol (28): (a) data collection, (b) data handling and linkage and (c) data visualization. All participant information, and data generated during this study was kept confidential in accordance with the HIPAA (Health Insurance Portability and Accountability Act of 1996) (36) on subject privacy and will not be used for any purpose other than conducting the study. Participant data was coded and matched to random numbers to ensure protection of data.

### Statistical Analysis

Descriptive statistics, including the observed frequency counts and percentages for healthcare professionals’ socio-demographic characteristics and cardiovascular risk factors included in the FBS [e.g., blood pressure (B), exercise (E), weight (W), alimentation (A), and tobacco (T)], were performed. Further, the Chi-square statistical test (at a significance level of 0.05) was applied to compare the distribution of responses to the gender. Following this, descriptive statistics (including means, standard deviations and ranges) were calculated for FBS and three MBI scales, namely emotional exhaustion (EE), personal accomplishment (PA) and depersonalization (DP). Further, a correlation analysis was carried out between FBS and all possible predictors, including MBI scales and socio-demographic information, to examine the statistical properties and test each coefficient’s significance using *p*-values.

Following the descriptive statistics, linear regression analysis was conducted to investigate the association between the outcome variable (FBS) and MBI scales adjusted with the socio-demographic factors. As socio-demographic information may be potential confounders of FBS, no variable selection approach (e.g., stepwise regression analysis) was applied. Considering potential effect of correlation among predictors, different models were created. To select the best fitting regression model, Akaike information criterion (AIC) was used as an estimator to compare the relative quality of statistical models. The variance inflation factor (VIF) diagnostic was also calculated in each model to prevent unreliable estimates of coefficients with possible high correlations among predictors. In the regression model, results are reported as coefficients with 95% confidence intervals (CIs) and *p* < 0.05 indicating statistical significance. Analyses were performed with STATA 16.1 (Stata Corp., LLC, United States).

## Results

By completing the online surveys, 537 healthcare professionals were recruited in the cohort. After cleaning up the data from incomplete or invalid questionnaires, the recruited cohort included 396 (73.7%) participants who completed socio-demographic characteristics, 396 (73.7%) who completed the MBIs’ subscale scores depersonalization (MBI-DP), 392 (73.0%) who completed personal accomplishment (MBI-PA), 389 (72.4%) who completed emotional exhaustion (MBI-EE), and 388 (72.2%) who completed the FBS. Of the 110 participants who used wearable sleep monitoring, 47 (42.73%) who had sleep reports that could be associated with the burnout and cardiovascular health scores were included in the study.

The socio-demographic characteristics of the participants are summarized and compared regarding the gender. As shown in Table 1A, it was noted that there is a difference in the distribution of responses to the gender among some comparison groups, such as age (*p* = 0.003), marital status (*p* = 0.002), specialty (*p* < 0.001), education (*p* < 0.001), and working hours (*p* = 0.025).

**TABLE 1A:** Participant socio-demographic characteristics.

Socio-demographic characteristics	Male	Female	Total	*P-*value
		
	*n* (%)	*n* (%)	*n* (%)	
**Age range, year**			396(100%)	0.003
< 30	17 (10.49)	46 (19.66)	63 (15.91)	
30–39	65 (40.12)	104 (44.44)	169 (42.68)	
40–49	50 (30.86)	65 (27.78)	115 (29.04)	
50 +	30 (18.52)	19 (8.12)	49 (12.37)	
**Marital status**			394(100%)	0.002
Single	19 (11.73)	50 (21.55)	69 (17.51)	
Married	142 (87.65)	172 (74.14)	314 (79.7)	
Others^a^	1 (0.62)	10 (4.31)	11 (2.79)	
**Specialty**			381(100%)	
Non-surgical specialty	55 (35.71)	76 (33.48)	131 (34.38)	< 0.001
Surgical specialty	12 (7.79)	4 (1.76)	16 (4.20)	
Nurses	22 (14.29)	101 (44.49)	123 (32.28)	
Allied health	31 (20.13)	15 (6.61)	46 (12.07)	
Others^b^	34 (22.08)	31 (13.66)	65 (17.06)	
**Education**			397(100%)	< 0.001
Diploma	7 (4.29)	40 (17.09)	47 (11.84)	
Bachelor	62 (38.04)	111 (47.44)	173 (43.58)	
Master	23 (14.11)	36 (15.38)	59 (14.86)	
PHD	5 (3.07)	6 (2.56)	11 (2.77)	
GP	13 (7.98)	11 (4.7)	24 (6.05)	
Specialist	23 (15.34)	20 (8.55)	45 (11.34)	
Consultant	28 (17.18)	10 (4.27)	38 (9.57)	
**Working hours**			395(100%)	0.025
< 30 h	9 (5.56)	10 (4.29)	19 (4.81)	
30–40 h	53 (32.72)	108 (46.35)	161 (40.76)	
> 40 h	100 (61.73)	115 (49.36)	215 (54.43)	

*^a^Others refer to divorced, widow and others.*

*^b^Others refer to participants who do not belong to one of the four specialties: non-surgical specialty, surgical specialty, nurses, allied health.*

Table 1B shows there is statistically significant difference in the distribution of responses to the gender among some FBS components, such as blood pressure (*p* < 0.001), exercise (*p* < 0.001), and tobacco (*p* < 0.001).

**TABLE 1B:** Fuster-BEWAT score (FBS): participant characteristics and descriptive statistics by gender (*N* = 388).

FBS components	Male	Female	Total	*P*-value
		
	*n* (%)	*n* (%)	*n* (%)	
Blood pressure (B)				< 0.001
[0] (SBP ≥ 140 and/OR DBP ≥ 90 MMHG)	8 (5.00)	5 (2.19)	13 (3.35)	
[1] (SBP 130–139 and/OR DBP 85–89 MMHG)	40 (25.00)	30 (13.16)	70 (18.04)	
[2] (SBP 120–129 and/OR DBP 80–84 MMHG)	63 (39.38)	66 (28.95)	129 (33.25)	
[3](SBP < 120 and DBP < 80 MMHG)	49 (30.63)	127 (55.70)	176 (45.36)	
Exercise [E]				< 0.001
[0] (< 10 moderate to vigorous activity min/week)	46 (28.75)	118 (51.75)	164 (42.27)	
[1] (< 75 moderate to vigorous activity min/week)	38 (23.75)	61 (26.75)	99 (25.52)	
[2] (75–149 moderate to vigorous activity min/week)	39 (24.38)	34 (14.91)	73 (18.81)	
[3] (≥ 150 moderate to vigorous activity min/week)	37 (23.13)	15 (6.58)	52 (13.40)	
Weight (W)				0.374
[0] (≥ 30 KG/M^2^)	33 (21.02)	35 (15.63)	68 (17.85)	
[1] (25 to < 30 KG/M^2^)	65 (41.40)	95 (42.41)	160 (41.99)	
[3] (< 25 KG/M^2^)	59 (37.58)	94 (41.96)	153 (40.16)	
Alimentation (A)				0.789
[0] (< 1 fruit/vegetable servings daily)	54 (33.96)	89 (38.86)	143 (36.86)	
[1] (1–2 fruit/vegetable servings daily)	65 (40.88)	86 (37.55)	151 (38.92)	
[2] (3–4 fruit/vegetable servings daily)	27 (16.98)	35 (15.28)	62 (15.98)	
[3] (> 4 fruit/vegetable servings daily)	13 (8.18)	19 (8.30)	32 (8.25)	
Tobacco (T)				< 0.001
[0] (> 1 pack of tobacco per day)	10 (6.25)	7 (3.04)	17 (4.36)	
[1] (< 1 pack of tobacco per day)	19 (11.88)	3 (1.30)	22 (5.64)	
[3] (non-smoker)	131 (81.88)	220 (95.65)	351 (90.00)	

Table 2 provides the descriptive results, while Table 3 shows the Spearman’s rank correlation coefficients for FBS, MBI scales and socio-demographic information.

**TABLE 2:** Descriptive statistics for fuster-bewat score (FBS), Maslach Burnout Inventory (MBI) emotional exhaustion (MBI-EE), depersonalization (MBI-DP), and personal accomplishment (MBI-PA) subscale scores.

Variable	Obs	Mean	*SD*	Min	Max
1. FBS	388	8.57	2.45	1	15
2. MBI-EE	389	23.53	15.18	0	54
3. MBI-PA	392	40.33	7.56	8	48
4. MBI-DP	396	6.39	6.72	0	30

**TABLE 3:** Spearman’s rank correlation coefficients for FBS, MBI dimensions and socio-demographic information (*p < 0.05).

Variables	(1)	(2)	(3)	(4)	(5)	(6)	(7)	(8)	(9)	(10)
(1) FBS	1.0									
(2) MBI-EE	−0.18*	1.0								
(3) MBI-PA	0.12*	−0.34*	1.0							
(4) MBI-DP	−0.11*	0.59*	−0.23*	1.0						
(5) Gender	0.04	0.20*	−0.12*	0.10	1.0					
(6) Age	0.04	−0.27*	0.16*	−0.27*	−0.17*	1.0				
(7) Marital status	0.07	–0.09	0.17*	−0.14*	–0.06	0.41*	1.0			
(8) Education level	0.04	0.04	–0.10	0.05	−0.31*	0.02	–0.04	1.0		
(9) Specialty	–0.03	–0.02	0.07	–0.09	–0.08	0.08	0.07	−0.15*	1.0	
(10) Working hours	–0.06	0.22*	–0.01	0.05	–0.09	0.07	0.08	0.02	0.05	1.0

*Fuster-BEWAT score (FBS), Maslach Burnout Inventory (MBI) emotional exhaustion (MBI-EE), depersonalization (MBI-DP), and personal accomplishment (MBI-PA) subscale scores.*

Statistically significant correlation has been observed among FBS, MBI components and some socio-demographic variables. In particular, high correlation among MBI components, such as between MBI-EE and MBI-DP (coef: 0.59, *p* < 0.05, Table 3).

Table 4 presents the results of the regression analysis with FBS as the outcome. Considering high correlations, different regression models were created to investigate the role of MBI components individually (model 1–3) and in combination with each other (model 4–7) adjusted with the socio-demographic factors. Seven models were built and AIC was employed to select the best fitting regression model.

**TABLE 4:** The regression models for Fuster-BEWAT score (FBS) with Maslach Burnout Inventory (MBI) emotional exhaustion (MBI-EE), depersonalization (MBI-DP), and personal accomplishment (MBI-PA) subscale scores (*N* = 388).

Model 1: FBS ∼ MBI-EE; adjusted for: gender, age, marital status, education level, specialty and working hours				
	**Predictor**	**Coef.**	**(95% CI)**	***p*-value**

	MBI-EE	–0.035	(−0.053, −0.017)	< 0.001**
	AIC_1_ = 1596.24			

**Model 2**: FBS ∼ MBI-PA; adjusted for: gender, age, marital status, education level, specialty and working hours				

	MBI-PA	0.061	(0.025, −0.097)	< 0.001**
	AIC_2_ = 1604.32			

**Model 3**: FBS ∼ MBI-DP; adjusted for: gender, age, marital status, education level, specialty and working hours				

	MBI-DP	–0.050	(−0.09, −0.01)	0.014*
	AIC_3_ = 1609.23			

**Model 4**: FBS ∼ MBI-EE and MBI-PA; adjusted for: gender, age, marital status, education level, specialty and working hours				

	MBI-EE	–0.029	(−0.048, −0.01)	0.002*
	MBI-PA	0.045	(0.007, 0.082)	0.019*
	Gender	0.61	(0.042, 1.17)	0.035*
	AIC_4_ = 1592.58			

**Model 5**: FBS ∼ MBI-PA and MBI-DP; adjusted for: gender, age, marital status, education level, specialty and working hours				

	MBI-PA	0.057	(0.020, 0.092)	0.002*
	MBI-DP	–0.043	(−0.082, −0.003)	0.035*
	AIC_5_ = 1601.73			

**Model 6**: FBS ∼ MBI-EE and MBI-DP; adjusted for: gender, age, marital status, education level, specialty and working hours				

	MBI-EE	–0.032	(−0.054, −0.011)	0.004*
	MBI-DP	–0.012	(−0.059, 0.036)	0.629
	AIC_6_ = 1598.01			

**Model 7**: FBS ∼ MBI-EE, MBI-PA and MBI-DP; adjusted for: gender, age, marital status, education level, specialty and working hours				

	MBI-EE	–0.026	(−0.048, −0.003)	0.025*
	MBI-PA	0.045	(0.008, 0.082)	0.018*
	MBI-DP	–0.014	(−0.061, 0.033)	0.567
	Gender	0.601	(0.034, 1.169)	0.038*
	AIC_7_ = 1594.25			

*Statistical significance, p < 0.05; ** high statistical significance, p < 0.01. Only statistically significant control variables (e.g., gender) was presented in the table. AIC: Akaike information criterion; CI: confidence interval.

Based on the AIC, the results suggested that Model 4 is the best fitting model (AIC_4_ = 1592.58). It showed a negative association between cardiovascular health and emotional exhaustion (*p* < 0.05). The higher the emotional exhaustion the lower the cardiovascular health. Further, the model showed a positive association between personal accomplishment and cardiovascular health (*p* < 0.05). It should be noted that Model 4 does not include depersonalization as a predictor. While Model 3 and 5 showed a statistically significant negative association between cardiovascular health and depersonalization (*p* < 0.01), Model 6 and 7 did not support this. However, such discrepancy for depersonalization effect may be explained by its monotonic relationship with emotional exhaustion (rho: 0.59, *p* < 0.05).

Emotional exhaustion was significantly positive correlated with REM and light sleep average (Spearman’s rank correlation: 0.35 and 0.37, respectively, with *P* < 0.05) (Table 5).

**TABLE 5:** Spearman’s rank correlation coefficients between Maslach Burnout Inventory, Fuster-BEWAT score (FBS), and sleep scores (light sleep average, REM sleep average, and long interruptions) (*N* = 47, *p < 0.05).

Variables	(1)	(2)	(3)	(4)	(5)	(6)	(7)
(1) FBS	1.000						
(2) MBI–EE	–0.287	1.000					
(3) MBI–PA	0.235	−0.427*	1.000				
(4) MBI–DP	–0.234	0.702*	−0.372*	1.000			
(5) LightsleepavgMin	–0.176	0.351*	–0.043	0.225	1.000		
(6) REMsleepavgMin	0.015	0.374*	–0.053	0.286	0.792*	1.000	
(7) Longinterruptin	–0.170	0.232	–0.115	0.250	0.223	0.245	1.000

## Discussion

As the study’s main finding, healthcare practitioners with a low composite cardiovascular health score are more emotionally exhausted. This is, to the best of our knowledge, the first prospective study in the Middle East that examines the relationship between burnout and cardiovascular health. Although this study cannot conclusively establish a causal relationship between burnout and cardiovascular risk, the data indicate that healthcare practitioners with burnout have an associated cardiovascular risk. Furthermore, preliminary data from this study highlights the relationship between emotional exhaustion and sleep alterations detected through heart rate variability monitoring, implying that sleep and heart rate variability measures could be a promising starting point for explaining the mechanisms underlying burnout symptoms and cardiovascular disease. The findings of this study may be relevant in creating preventive strategies for burnout and cardiovascular risk reduction or prevention.

In this study, some comparison groups have different distributions of responses to gender, such as age (more women in the preponderant age group of 30–39), marital status (higher rate of single women than men, married men than women), specialty (more women than men nurses), working hours (more women than men in the category with 30–40 h per week, and more men than women in the category of more than 40 h per week) and education (more women than men in the preponderant group with bachelor degree). Professional specialty was found to be significantly related to high levels of emotional exhaustion and depersonalization among hospital healthcare workers during the COVID-19 pandemic (37, 38). The groups with a higher response rate in this study may indicate that they are more receptive to psychological support programs. These findings corroborate and complement a study that examined the impact of the COVID-19 outbreak on health professionals in Northern Italy and identified female gender, nurse status, hospital work, and contact with COVID-19 patients as predictors of both emotional exhaustion and depersonalization (39). The disparities in response distribution in our study, on the other hand, could point to a general distribution per category that is independent of the pandemic. For example, the fact that more men reported high blood pressure and smoking while more women reported low physical activity may reflect the well-known high prevalence of hypertension and smoking in men while women are more sedentary (40, 41).

A high correlation between the dimensions of burnout-Emotional Exhaustion (EE), Depersonalization (DP) has been observed in this study. Fear of COVID-19 infection was linked to extreme emotional exhaustion and depersonalization (37). This study is consistent with a study of Bulgarian healthcare professionals, which found that emotional exhaustion and depersonalization are associated with low levels of sense of coherence (SOC) as a salutogenic construct in three dimensions: meaningfulness (Me), the desire of an individual to be motivated to cope; comprehensibility (C), the belief that the challenge is understood; and manageability (Ma), the belief that coping resources are available (42).

A negative association between cardiovascular health and emotional exhaustion has been observed in this study. The higher the emotional exhaustion the lower the cardiovascular health. Further, the model showed a positive association between personal accomplishment and cardiovascular health. Recent research indicates that a variety of psychosocial risk factors, such as work stress (43), vital exhaustion (44), or social isolation (45), raise the risk of recurrent cardiac events as well as cardiac and all-cause mortality. Screening for such psychosocial risk factors in populations with cardiovascular risk factors has the potential to improve understanding of their role in the occurrence and outcome of cardiovascular disease (46).

This study establishes a link between emotional exhaustion and REM sleep periods in healthcare professionals during the COVID-19 pandemic. Sleep, and particularly REMS, have recently been shown to play an important role in the emotional and mental recovery from difficult circumstances (47). A positive relationship between exhaustion and insomnia has been already observed in healthcare professionals already in March and April 2020 during the COVID-19 pandemic (48). Sleep quality can be influenced by high psychological distress, high emotional exhaustion, low depersonalization, and low personal accomplishment (49). Furthermore, sleep disturbances have been linked to psychological distress in healthcare workers during the COVID-19 pandemic (50). Sleep disturbances are increasingly being linked to the development of cardiovascular diseases. The Sleep Heart Health Study, which monitored 3,810 participants for 11 years, revealed that inefficient sleep was related with an increased risk of incident cardiovascular disease events (51). Sleep disturbances have been linked to an increased risk of cardiovascular disease. Heart rate variability, a measure of autonomic nervous system dysfunction seen in sleep disorders, appears to be an essential marker of this risk (52–54). On the mechanisms that may be responsible for these sleep changes, the heart rate variability that is at the heart of the algorithm used to describe these sleep stages is also considered to be a reflection of the autonomic nervous system tone (55, 56). In this line of evidence, a recent study demonstrated that vagal dysfunction is both predictive and specific for burnout symptoms, suggesting that heart rate variability may be a promising starting point for explaining the mechanisms underlying burnout symptoms and cardiovascular diseases (57). Wearable sleep monitoring is gaining popularity, indicating the possibility of expanding burnout research through the use of wearable technology (58). On the other hand, the wide range of accuracy among commercial sleep technologies highlights the critical need for ongoing evaluations of newly developed sleep technologies (59, 60).

### Study Limitations

The study’s shortcomings must be acknowledged. The main weakness of this cross-sectional study was its inability to assess incidence and draw causal conclusions due to its design. There is no evidence of a temporal link between exposure and result. Although this study was originally intended to be a longitudinal one, it was converted to a cross-sectional one due to pandemic restrictions that made onsite visits difficult (28). As a result, the number of participants using wearable technology was significantly lower when compared to data gathered from online surveys. Another reason for avoiding the use of wearable technology was apprehension about being tracked by a GPS device. Another important factor that contributed to a decrease in interest for both recruiters and participants in participating in onsite visits was the longer-than-expected duration of social restrictions caused by pandemics.

## Conclusion

In conclusion, this study provides evidence for a link between burnout and cardiovascular risk. Sleep disturbances caused by an altered autonomic nervous system may represent a mechanistic link between burnout and cardiovascular disease. These findings may lend support for policymakers and organizations campaigning for better occupational health. The development of occupational health surveillance and workplace health promotion initiatives by policymakers to prevent and treat burnout as well as other mental health disorders in the workplace during and after the COVID 19 pandemic is a potential future direction (61).

## Data Availability Statement

The datasets generated during and/or analyzed during the current study are available from the corresponding author on reasonable request.

## Ethics Statement

Ethical approval was obtained from the Abu Dhabi COVID19 Research IRB Committee of the Department of Health-Abu Dhabi (DOH/NCVDC/2020/1052) and Emirates IRB for COVID Research Committee (DOH/CVDC/2020/1246). The patients/participants provided their written informed consent to participate in this study.

## Author Contributions

HA, OB, MA, MD, WA, and LC: study conception and design. FA, NA, SA, MB, NA, MA, and MD: investigation. MS, NU, EA, LC, HA, and OB: data curation and analysis. FA, NA, SA, MB, NA, MS, NU, MD, MA, LC, WA, EA, HA, and OB: interpretation of the data, writing of the manuscript, and critical revision of the manuscript regarding the important intellectual content. All authors contributed to the article and approved the submitted version.

## Conflict of Interest

The authors declare that the research was conducted in the absence of any commercial or financial relationships that could be construed as a potential conflict of interest.

## Publisher’s Note

All claims expressed in this article are solely those of the authors and do not necessarily represent those of their affiliated organizations, or those of the publisher, the editors and the reviewers. Any product that may be evaluated in this article, or claim that may be made by its manufacturer, is not guaranteed or endorsed by the publisher.
